# Supplementary material for: Dietary Supplements during COVID-19 Outbreak. Results of Google Trends Analysis Supported by PLifeCOVID-19 Online Studies
Source: Nutrients. 2020 Dec 27;13(1):54. doi: 10.3390/nu13010054 (PMC7823317; doi:10.3390/nu13010054)
Supplement: Supplementary file 1 [file nutrients-13-00054-s001.pdf]

nutrients MDPI

Supplementary Materials

Table S1. Checklist for documentation of Google Trends (based on Nuti et al. [20] and Kamiński et al. [16]).

Section/topic	Checklist item
**Search variables**	
Access Date	2 November 2020
Time Period	From 1 January 2020 to 31 October 2020
Query Category	Health
Region	Worldwide and Poland
**Search Input**	
Non-adjusted	Topic "coronavirus" and topics related to immunity, dietary supplements and nutrients, bioactive compounds and plants traditionally used to boost the immunity: "Immune system", "dietary supplement", "Vitamins", "Mineral", "Vitamin D", "Vitamin K", "Vitamin C", "Vitamin A", "Vitamin B_{12}", "Zinc", "Iron", "Iodine", "Selenium", "Magnesium", "Copper", "Omega-3 fatty acids", "Fish oil", "Probiotic", "Rutin", "Acerola", "Garlic", "Onion", "Ginger", "Turmeric", "Echinacea", "Lactoferrin", "Ashwagandha", "Honey", "Elderberry", "Raspberry", "Sea buckthorn", "Nigella sativa", "Glycyrrhiza glabra"
Adjusted	Topic "Lutein" + separately all remain topics
**Rationale for Search Strategy**	
For Search Input	The searched topics are related to immune system and nutrients, bioactive compounds or foods linked to immune system. We compared relative search volumes of all topics with topic "Lutein", because of the possibility of comparing only five items at one time in the Google Trends. Due to this we could compare all queries in further analysis.
For Setting Chosen	We choose the "health" query category because we wanted to assess the interest in the context of health, whereas some of topics may be related to industry, gardening or culinary.

Table S2. Top five rising queries related to search term worldwide and in Poland.

Topic	Worldwide	Poland
Coronavirus	Coronavirus update	Coronavirus symptoms
	Coronavirus UK	Symptoms of coronavirus
	News coronavirus	Coronavirus map
	Coronavirus map	Poland coronavirus
	USA coronavirus	Coronavirus in Poland
Immune system	Corona immunity	Immune system
	Immune to coronavirus	
	Are you immune to coronavirus after having it	
	once you get coronavirus are you immune	
Dietary supplements	Penis increase supplement	Cla
	Resurge supplement	Dietary supplement and drugs
	Lactoferrin supplement	Msm supplements
	Quinine supplement	Dietary supplements on weight loss
	Supplement group	4her supplements
Vitamins	Vitamin D covid	Vitamin for free
	Vitamin D coronavirus	Coronavirus vitamin C
	Vitamin D covid 19	Vitamin D for free
	Vitamin C coronavirus	Vitamin D3 for free
	Vitamin d and covid	Vitamin hsdi (hygiene, sleep, diet and isolation)
Minerals	calcium and magnesium mineral	Minerals
	mineral mind map	Vitamins and minerals
	what are the nutrients our body needs	Schnidele's minerals
	kelat vitamins and minerals	
	humic minerals	
Vitamin D	Vitamin D covid	Vitamin D3 for free
	Vitamin D coronavirus	Vitamin D on coronavirus
	Covid 19 vitamin D	Vitamin D for free
	Coronavirus vitamina d	Sources of vitamin D
	Vitamin d and covid	Best vitamin D3 drug
Vitamin K	Vitamin K Covid	Konakion
	Vitamin K coronavirus	

	Corona vitamin K<br>Foods with vitamin K<br>Vitamin K tablets	
Vitamin C	Coronavirus vitamin C<br>Vitamin C covid<br>Corona vitamin C<br>Coronavirus vitamin C<br>Vitamin C for coronavirus	Vitamin C on coronavirus<br>Best sources of vitamin C<br>Ascorvita<br>Vitamina c 2000<br>Rutioscorbin vitamin C forte
Vitamin A	Vitamin A<br>foods with vitamin A<br>retinol ergocalciferol what is it for<br>mad hippie vitamin a serum<br>retinol ergocalciferol	Retinol<br>Vitamin A
Vitamin B_{12}	thiaminal b12 price<br>What is thiamine pyridoxine cyanocobalamin for<br>hydroxyl b1 b6 b12<br>xedenol b12<br>What does vitamin B12 do?	Vitamin B12 drug<br>Vitamin B12 sources<br>Vitamin B12<br>Cobalamin<br>B12 forte
Zinc	Coronavirus zinc<br>Hudroxychloroquine zinc<br>Zinc and coronavirus<br>Zinc for coronavirus<br>Zithromax	Zinc and selenium<br>Zinc<br>Selenium zinc
Iron	Oxygen iron berry<br>Iron skin potion<br>Floradix ijzer	Sources of iron<br>Best sources of iron<br>Iron supplementation<br>High level of iron<br>Iron sources
Iodine	Does iodine kill viruses<br>povidone iodine prep pad	Iodine in drops
Selenium	Selenium coronavirus<br>what is selenium in<br>What chemicals are released into the environment when you smoke a cigarette<br>Selenium benefits	Selenium and thyroid<br>Selenium in diet<br>Brazil nut<br>Brazil nut selenium

	Selenium and coronavirus	Organic selenium
Omega-3 fatty acids	möllers omega 3 price<br>möllers omega 3<br>omega-3 supplements<br>omega-3 fatty acids foods<br>foods with omega 3	Kfd omega 3<br>Solgar omega 3<br>Omega 3 6 9<br>Evening primrose oil<br>Gold omega 3
Fish oil	Pristine fish oil<br>Best fish oil supplement 2020<br>The benefits of fish oil for cats<br>How to eat fish oil<br>Vascepa vs fish oil	Fish oil Pan Tabletka<br>Fish oil hasco+<br>Fish oil on immunity<br>Fish oil in capsules
Probiotic	c23 probiotics<br>ps128 Spiritual Probiotics<br>Pro b fresh<br>Pro b fresh dental probiotics<br>Dental probiotics uk	Miya probiotic serum<br>Latopic probiotics<br>Probiotic on guts<br>San probi<br>Swanson probiotic
Rutin	E rutin<br>Ingredients rich in rutin<br>Rutinoscrorbin<br>Rutoside<br>trypsin bromelain and rutoside trihydrate tablets uses	Rutin c
Acerola	one acerola equals how many oranges<br>what is acerola leaf tea for<br>acerola 1000<br>vitamine c naturelle<br>acerola tea	Vitamin C acerola<br>Acerola
Garlic	Garlic coronavirus<br>Red garlic<br>Garlic on viruses<br>Antiviral garlic<br>How to plant garli	Garlic on viruses<br>Blooming garlic<br>Garlic chives<br>When to plant winter garlic<br>Garlic health benefits
Onion	Salmonella outbreak<br>Salmonella outbreaks onion<br>Onions salmonella recall	Onion on viruses<br>Onion garlic syrup<br>Onion syrup on which cough

	Onion coronavirus<br>Red onion salmonella	Is the onion healthy<br>Onion juice
Ginger	black ginger syrup<br>black ginger<br>ginger on viruses<br>red ginger bejo<br>What is ginger with lemon for?	Ginger on viruses<br>Is ginger lower blood pressure<br>Turmeric<br>Syrup from lemon, ginger and honey<br>Is ginger raises blood pressure
Turmeric	plants containing curcuma<br>cod liver oil with turmeric<br>what is turmeric for<br>is turmeric antiviral<br>lakadong turmeric	Turmeric benefits<br>Turmeric on weight loss<br>Curcumin<br>Turmeric root<br>Turmeric tablets
Echinacea	Coronavirus echinacea<br>Echinacea covid<br>Echinacea corona<br>Echinacea covid 19<br>Equinacea coronavirus	Echinacea<br>Echinacea tablets
Lactoferrin	Lactoferrin covid<br>Pure lactoferrin<br>Lactoferrin coronavirus	Lactoferrin price
Ashwagandha	ashvaganda effects<br>resurge<br>ashwagandha gummies<br>how to boost testosterone	Ashwagandha what is it
Honey	Honey pot suppositories<br>Almond milk honey flat white<br>Zestmag honey prices at pharmacies<br>Honey under the microscope<br>Zestmag honey	Honey Minecraft<br>Honey price 2020<br>Honey prices 2020<br>Wood's honey benefits<br>The use of goldenrod's honey
Elderberry	Elderberry coronavirus<br>Elderberry and coronavirus<br>Elderberry for coronavirus<br>Cytokine storm<br>Elderbery cytokine storm	Elderberry benefits

Raspberry	Raspberry oil<br>Raspberry extract capsules<br>Raspberry vitamins<br>Raspberry ketone review uk	Raspberry kcal<br>Raspberry honey<br>Raspberry diseases<br>Raspberry juice
Sea buckthorn	sea buckthorn for pregnant<br>Sea buckthorn Vestige<br>Useful properties of sea buckthorn<br>Sea buckthorn oil	-
Nigella sativa	Black seed oil coronavirus<br>Chloroquine nigella<br>Full spectrum black seed oil<br>The Prophet's hadith about the black seed<br>Black seed to strengthen the immune system	Black seed cultivation<br>Black seed what for<br>Black seed<br>Black seed capsules<br>Nigella sativa
Glycyrrhiza glabra	Licorice root benefits<br>licorice coronavirus<br>licorice root<br>Root of licorice<br>Licorice	Licorice coronavirus<br>Licorice root benefits<br>Licorice root<br>Root of licorice

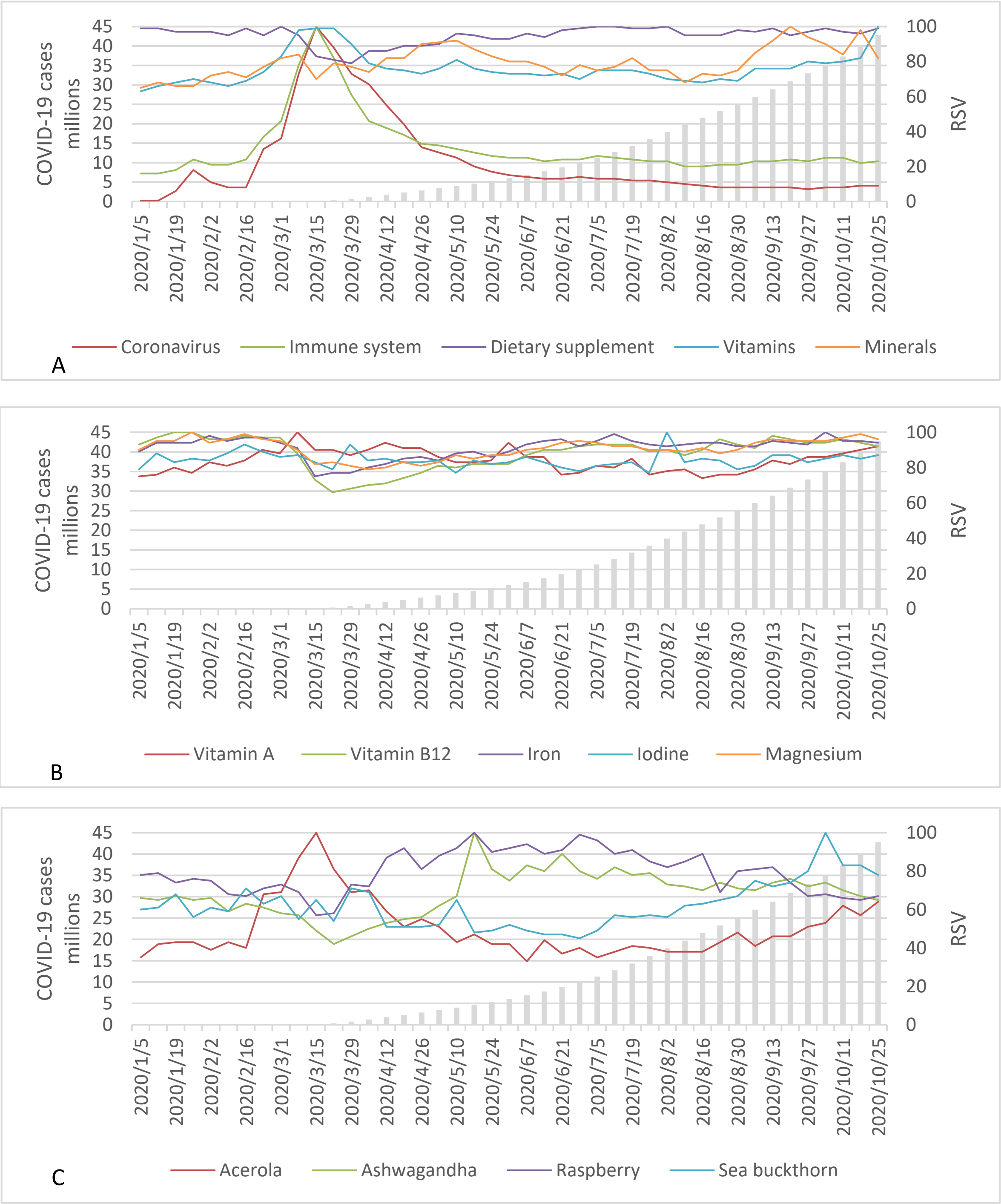

Figure S1. Trend curves of RSVs for nutrients, bioactive compounds and herbs search queries and cumulative confirmed COVID-19 cases in the World between 01 January and 31 October 2020. (A) coronavirus, immune system, dietary supplement, vitamins, minerals; (B) vitamins A, B_{12}, iron, iodine, magnesium; (C) acerola, ashwagandha, raspberry, sea buckthron.

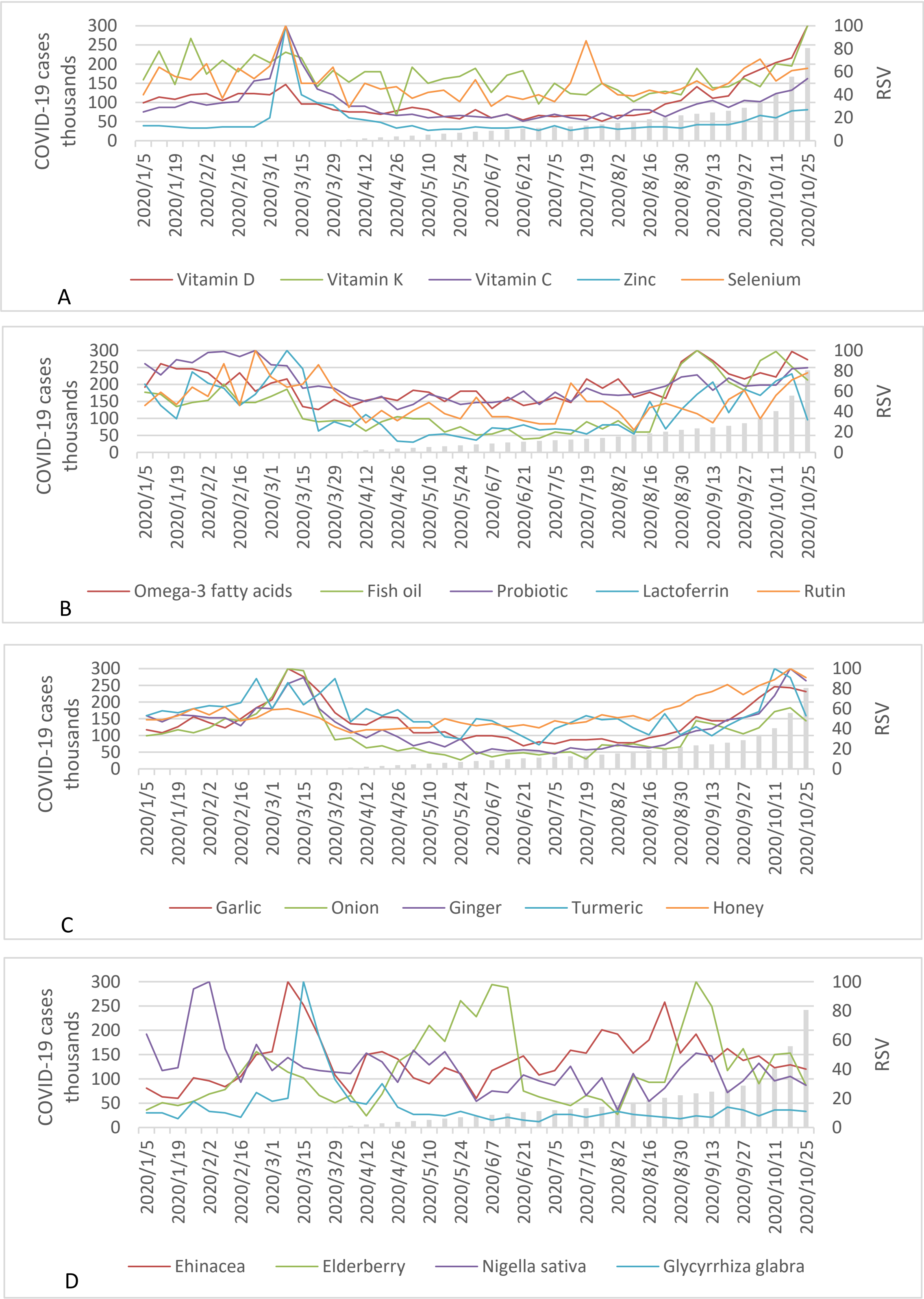
COVID-19 cases thousands
300
250
200
150
100
50
0
RSV
100
80
60
40
20
0
2020/1/5
2020/1/19
2020/2/2
2020/2/16
2020/3/1
2020/3/15
2020/3/29
2020/4/12
2020/4/26
2020/5/10
2020/5/24
2020/6/7
2020/6/21
2020/7/5
2020/7/19
2020/8/2
2020/8/16
2020/8/30
2020/9/13
2020/9/27
2020/10/11
2020/10/25
Vitamin D
Vitamin K
Vitamin C
Zinc
Selenium
A
Omega-3 fatty acids
Fish oil
Probiotic
Lactoferrin
Rutin
B
Garlic
Onion
Ginger
Turmeric
Honey
C
Ehinacea
Elderberry
Nigella sativa
Glycyrrhiza glabra
D

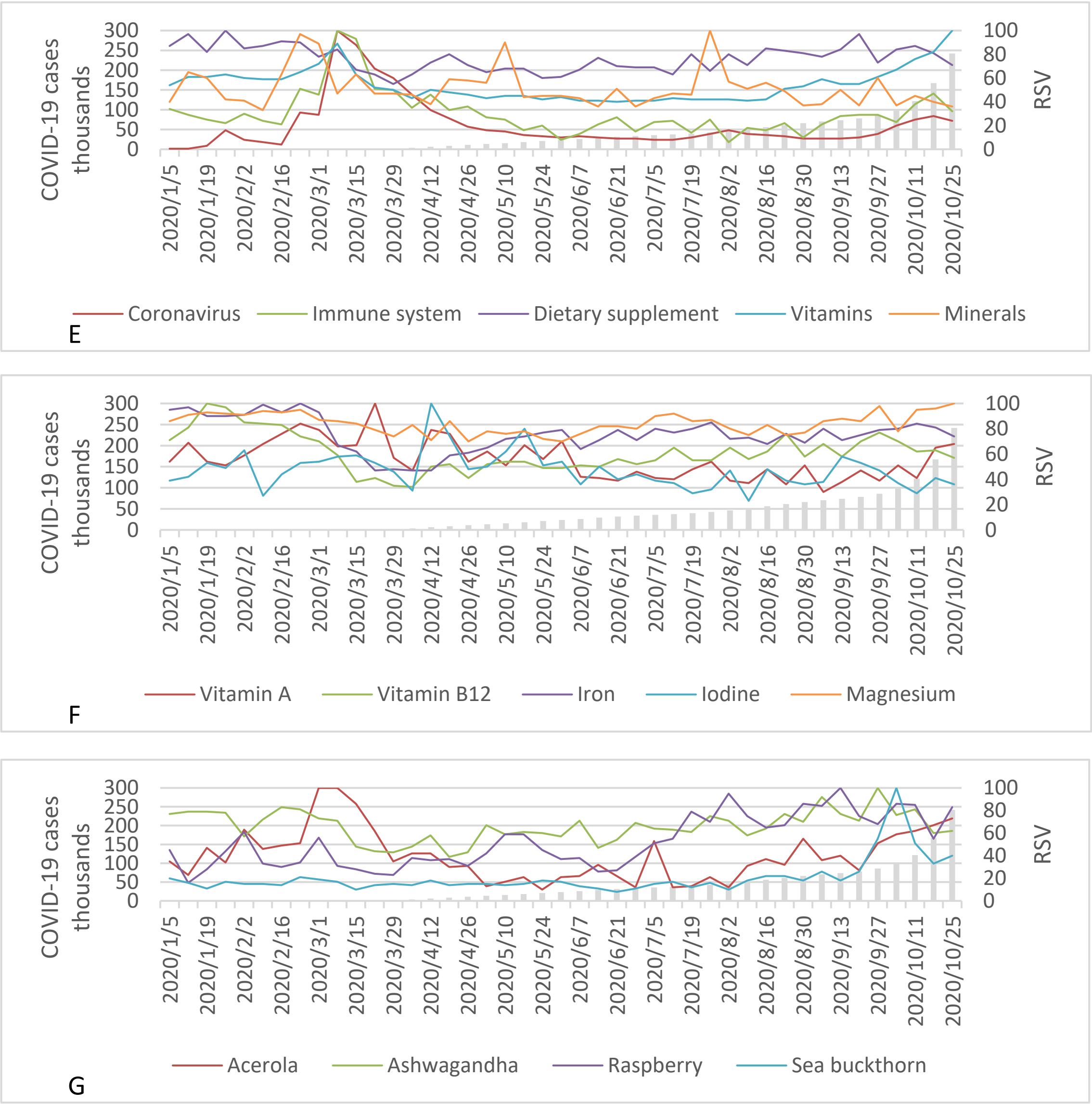

Figure S2. Trend curves of RSVs for nutrients, bioactive compounds and herbs search queries and cumulative confirmed COVID-19 cases in the Poland between 01 January and 31 October 2020. (A) vitamins D, K, C, zinc, selenium; (B) omega-3 fatty acids, fish oil, probiotics, lactoferrin, rutin; (C) garlic, onion, ginger, turmeric, honey; (D) echinacea, elderberry, *Nigella sativa*, *Glycyrrhiza glabra*; (E) coronavirus, immune system, dietary supplement, vitamins, minerals; (F) vitamins A, B_{12}, iron, iodine, magnesium; (G) acerola, ashwagandha, raspberry, sea buckthron.

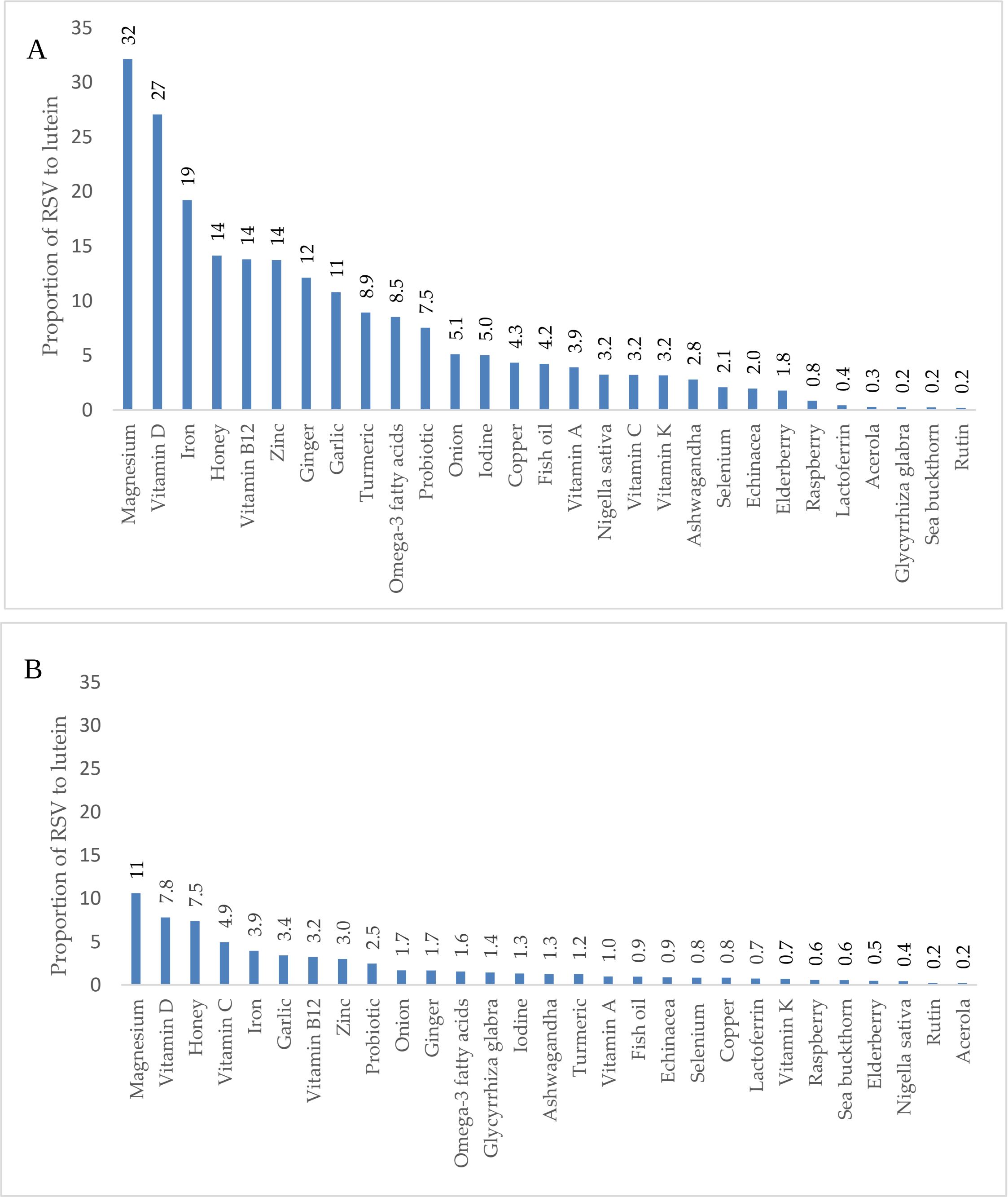

Figure S3. Popularity of nutrients, bioactive compounds and foods in proportion to "lutein" (A) worldwide and (B) in Poland between 01.01.2020 and 31.10.2020.

Table S3. Top five countries and place of set of Poland in search topics related to immune system and dietary supplements.

Topic	Countries
Coronavirus	Ethiopia (100), Qatar (86), Nepal (84), Italy (67), Spain (65), P**oland (10, no. 62)**
Immune system	Ghana (100), Lebanon (80), Ecuador (71), Indonesia (71), Philippines (71), **Poland (<0.1, no. 56)**
Dietary supplement	Japan (100), Italy (76), USA (62), Australia (60), South Africa (60), **Poland (26, no. 33)**
Vitamins	Iran (100), Saudi Arabia (88), Syria (78), El Salvador (75), Puerto Rico (73), **Poland (26, no. 35)**
Mineral	Iran (100), Thailand (88), Ghana (66), El Salvador (64), Mexico (61), **Poland (39, no. 17)**
Vitamin D	Libya (100), Iraq (68), Jordan (61), Azerbaijan (60), Saudi Arabia (56), **Poland (43, no. 29)**
Vitamin K	Bolivia (100), The Netherlands (98), Honduras (70), Venezuela (68), Guatemala (67), **Poland (35, no. 34)**
Vitamin C	**Poland (100)**, Brazil (74), Hungary (72), Italy (71), The Netherlands (35),
Vitamin A	Bolivia (100), Iran (93), Nepal (88), Mexico (86), Guatemala (83), **Poland (46, no. 31)**
Vitamin B_{12}	Syria (100), Lebanon (92), Jordan (86), The Netherlands (81), Turkey (75), **Poland (41, no. 31)**
Zinc	Iran (100), Iraq (32), Serbia (31), Bolivia (29), Syria (25), **Poland (11, no. 36)**
Iron	Vietnam (100), Jordan (92), Australia (69), Kuwait (66), Iran (66), **Poland (32, no. 32)**
Iodine	Albania (100), Kosovo (39), Philippines (12), North Macedonia (10), Serbia (9), **Poland (4, no. 18)**
Selenium	Serbia (100), Montenegro (81), Kosovo (39), Bosna and Herzegovina (37), Albania (30), **Poland (6, no. 18)**
Magnesium	Hungary (100), Moldova (79), Albania (76), Morocco (76), Bosna and Herzegovina (74), **Poland (39, no. 22)**
Omega-3 fatty acids	Bolivia (100), Honduras (84), Georgia (81), Puerto Rico (80), Kazakhstan (75), **Poland (32, no. 65)**
Fish oil	Taiwan (100), Turkey (71), Malaysia (62), Singapore (54), Denmark (49), **Poland (26, no. 33)**
Probiotic	Taiwan (100), Hong Kong (50), Serbia (38), Bosna and Herzegovina (35), Slovakia 32), **Poland (24, no. 14)**
Rutin	**Poland (100)**, Czech Republic (65), Lithuania (65), Uzbekistan (64), Russia (59)
Acerola	Morocco (100), North Macedonia (97), Martinique (94), Brazil (60), **Poland (43)**
Garlic	Jamaica (100), Nigeria (96), Ghana (96), Indonesia (92), Cameroon (75), **Poland (32, no. 33)**
Onion	Turkey (100), Serbia (86), Iran (76), Azerbaijan (70), Bolivia (66), **Poland (36, no. 27)**
Ginger	Ghana (100), Indonesia (91), Nigeria (86), Zambia (84), Cameroon (69), **Poland (13, no. 64)**
Turmeric	Jamaica (100), Ghana (97), Nigeria (81), Trinidad and Tobago (72), Kenya (62), **Poland (13, no. 56)**
Echinacea	Bolivia (100), Puerto Rico (86), Switzerland (54), Guatemala (48), Uruguay (44), **Poland (30, no. 12)**
Lactoferrin	Italy (100), Egypt (41), **Poland (23)**, Albania (17), Japan (15)
Ashwagandha	India (100), Trinidad and Tobago (98), Nepal (77), **Poland (75)**, United Arab Emirates (66),
Honey	Indonesia (100), Yemen (66), Libya (64), Cameroon (48), Saudi Arabia (48), **Poland (35, no. 13)**
Elderberry	Romania (100), USA (38), Moldova (33), Slovenia (33), Puerto Rico (31), **Poland (18, no. 12)**

Raspberry	**Poland (100)**, Palestine (95), UK (84), Australia (78), Canada (73),
Sea buckthorn	Slovakia (100), Moldova (91), Hungary (80), Latvia (79), Azerbaijan (74), **Poland (58, no. 11)**
Nigella sativa	Iran (100), Afghanistan (67), Nigeria (53), Ghana (46), Libya (45), **Poland (5, no. 45)**
Glycyrrhiza glabra	Turkmenistan (100), Kirgizstan (86), **Poland (84)**, Azerbaijan (83), Kazakhstan (82)

Table S4. Spearman rank's coefficients between nutrients, bioactive compounds and herbs RSVs and cumulative confirmed COVID-19 cases, deaths and Coronavirus RSV worldwide and in Poland.

Search query	Worldwide			Poland		
	Cumulative COVID-19 cases	Cumulative COVID-19 deaths	"Coronavirus" RSV	Cumulative COVID-19 cases	Cumulative COVID-19 deaths	"Coronavirus" RSV
Immune system	-0.22	-0.22	0.87***	-0.31*	-0.32*	0.55***
Dietary supplement	0.23	0.23	-0.55***	-0.09	-0.09	-0.26
Vitamins	0.28	0.28	0.48**	-0.13	-0.14	0.26
Mineral	0.50***	0.50***	0.14	-0.29	-0.26	0.19
Vitamin A	-0.06	-0.06	0.57***	-0.60***	-0.60***	0.31*
Vitamin B_{12}	0.02	0.02	-0.69***	-0.07	-0.06	-0.44**
Iron	0.22	0.22	-0.55***	-0.16	-0.14	-0.49***
Iodine	-0.09	-0.08	-0.09	-0.39**	-0.40**	0.24
Magnesium	0.18	0.18	-0.60***	-0.02	-0.02	-0.18
Copper	0.33*	0.33*	-0.06	0.01	0.01	0.20
Acerola	-0.07	-0.07	0.44	-0.08	-0.09	0.32*
Lactoferrin	0.71***	0.71***	-0.21	-0.11	-0.12	0.10
Ashwagandha	0.52***	0.52***	-0.37*	0.08	0.08	-0.34*
Raspberry	0.05	0.05	0.18	0.72***	0.72***	-0.07
Sea buckthorn	0.32*	0.32*	-0.47**	0.45**	0.45**	0.20

RSV – Relative Search Volume; * $p \leq 0.05$; ** $p \leq 0.01$; *** $p \leq 0.001$.

Table S5. Study group characteristic according to the use of dietary supplements during COVID-19.

Variables	PLifeCOVID-19 Online Study 1			PLifeCOVID-19 Online Study 2		
	Total	Dietary supplement users	p-Value	Total	Dietary supplement users	p-Value

	100% (n = 2296)	Before pandemic 34% (n = 773)	During pandemic 14% (n = 316)	No 53% (n = 1207)		100% (n = 978)	Before pandemic 45% (n = 436)	During pandemic 34% (n = 334)	No 21% (n = 208)	
**Gender:**										
female	91 (2078)	94 (728)	94 (296)	87 (1054)	<0.001	94 (916)	97 (423)	95 (317)	85 (176)	<0.001
male	9.5 (218)	5.8 (45)	6.3 (20)	13(153)		6.3 (62)	3.0 (13)	5.1 (17)	15 (32)	
**Age:**										
<30 years	30 (690)	33 (252)	34 (106)	28 (332)		24 (231)	21 (91)	22 (73)	32 (67)	
30-39 years	46 (1051)	48 (370)	41 (128)	46 (553)		58 (565)	63 (275)	59 (198)	44 (92)	
40-49 years	12 (282)	11 (85)	13 (42)	13 (155)	0.002	8.1 (79)	7.3 (32)	9.3 (31)	7.7 (16)	<0.001
50-59 years	6.3 (144)	4.5 (35)	5.1 (16)	7.7 (93)		5.3 (52)	4.4 (19)	5.4 (18)	7.2 (15)	
≥60 years	5.5 (127)	4.0 (31)	7.6 (24)	6.0 (72)		5.2 (51)	4.4 (19)	4.2 (14)	8.7 (18)	
**Education level:**										
lower	22 (505)	17 (129)	22 (69)	25 (307)	<0.001	18 (171)	12 (54)	12 (39)	38 (78)	<0.001
higher (university)	78 (1791)	83 (644)	78 (247)	75 (900)		83 (807)	88 (382)	88 (295)	63 (130)	
**Housing situation:**										
living alone	9.1 (208)	9.2 (71)	12 (37)	8.3 (100)		3.6 (35)	2.8 (12)	3.6 (12)	5.3 (11)	
living with partner	22 (496)	22 (168)	30 (94)	19 (234)		13 (124)	12 (52)	15 (50)	11 (22)	
living with partner and/or children	58 (1330)	59 (456)	47 (147)	60 (727)	<0.001	68 (661)	73 (316)	68 (226)	57 (119)	<0.001
living with parents or other relatives	11 (262)	10 (78)	12 (38)	12 (146)		16 (158)	13 (56)	1.8 (46)	27 (56)	
**Place of living:**										
rural	16 (363)	14 (107)	12 (38)	18 (218)		19 (188)	17 (73)	16 (54)	29 (61)	
town <50,000 inhabitants	16 (373)	15 (117)	16 (49)	17 (207)		16 (156)	15 (66)	14 (48)	20 (42)	
town 50,000-100,000 inhabitants	11 (262)	10 (80)	10 (32)	12 (150)	0.02	9.3 (91)	8.9 (39)	11 (35)	8.2 (17)	0.002
town 101,000-500,000 inhabitants	14 (327)	15 (112)	18 (56)	13 (159)		16 (156)	16 (71)	17 (57)	14 (28)	
town >500,000 inhabitants	16 (362)	17 (133)	17 (54)	15 (175)		17 (170)	18 (79)	19 (64)	13 (27)	
urban agglomeration	27 (609)	29 (224)	28 (87)	25 (298)		22 (217)	25 (108)	23 (76)	16 (33)	

**Macroeconomic region:**										
<50% of EU-28 GDP	17 (378)	15 (116)	14 (44)	19 (223)		16 (152)	12 (53)	14 (46)	26 (53)	
50-100% of EU-28 GDP	60 (1344)	61 (474)	63 (198)	59 (711)	0.1	68 (660)	71 (310)	68 (228)	59 (122)	<0.001
>100% of EU-28 GDP	23 (521)	24 (183)	23 (74)	23 (273)		17 (166)	17 (73)	18 (60)	16 (33)	
**Employment forms during pandemic:**										
did not work or considerable work time reduction	45 (1023)	52 (405)	36 (113)	42 (505)		28 (275)	33 (145)	26 (86)	21 (44)	
began remote work and/or study	41 (946)	39 (298)	50 (158)	41 (490)	<0.001	37 (365)	35 (152)	40 (135)	38(78)	0.02
work in the same form as earlier	14 (327)	9.1 (70)	14 (45)	18 (212)		35 (338)	32 (139)	34(113)	41(86)	
**BMI before pandemic:**										
underweight	6.0 (138)	7.4 (57)	5.4 (17)	5.3 (64)		4.8 (47)	5.5 (24)	3.3 (11)	5.8 (12)	
normal weight	59 (1344)	63 (484)	64 (202)	55 (722)	<0.001	60 (591)	62 (269)	62 (207)	55 (115)	0.5
overweight	25 (582)	21 (164)	26 (83)	28 (335)		25 (242)	23 (100)	25 (82)	29 (60)	
obesity	10 (232)	8.8 (68)	4.4 (14)	12 (150)		10 (98)	9.9 (43)	10 (34)	10 (21)	

BMI – body mass index; GDP – gross domestic product.

Table S6. Nutrients and bioactive compounds supplemented during COVID-19 pandemic – results of PLifeCOVID-19 Online Study 1 and 2.

Nutrients	PLifeCOVID-19 Online Study 1		PLifeCOVID-19 Online Study 2		
	**Supplemented during pandemic**	**Supplementation started during pandemic**	**Supplemented during pandemic**	**Supplementation started during first wave of pandemic**	**Supplementation started during second wave of pandemic**
Vitamins B	13 (307)	1.8 (41)	33 (323)	9.4 (92)	1.6 (16)
Vitamin B_{12}	11 (263)	1.4 (32)	25 (245)	7.2 (70)	0.8 (8)
Folic acid	15 (336)	1.7 (39)	32 (311)	7.9 (77)	0.9 (9)
Biotin	6.2 (142)	1.4 (33)	12 (121)	3.5 (34)	0.7 (7)
Vitamin A	9.9 (227)	2.6 (60)	22 (217)	7.7 (75)	1.3 (13)
Vitamin E	11 (241)	2.6 (60)	23 (225)	7.4 (72)	0.9 (9)
Choline	2.7 (61)	0.1 (3)	4.2 (41)	1.4 (14)	0 (0)
Iron	12 (279)	1.5 (35)	26 (249)	5.8 (57)	0.6 (6)
Iodine	10 (232)	1.0 (23)	26 (251)	5.9 (58)	0.5 (5)
Magnesium	15 (334)	2.0 (46)	23 (220)	5.2 (51)	1.6 (16)
Calcium	4.4 (101)	0.7 (15)	12 (118)	3.7 (36)	0.7 (7)
Potassium	1.5 (36)	0.2 (6)	3.1 (30)	1.2 (12)	0.1 (1)
Chrome	1.4 (32)	0.5 (12)	2.4 (23)	1.1 (11)	0.1 (1)
Manganese	2.5 (57)	0.6 (14)	4.9 (48)	1.5 (15)	0.4 (4)
Copper	3.7 (86)	0.8 (19)	8.2 (80)	1.9 (19)	0.4 (4)
Molybdenum	1.8 (41)	0.4 (9)	3.5 (34)	0.9 (9)	0.3 (3)
Silicon	0.4 (11)	0.4 (9)	2.7 26)	1.5 (15)	0.1 (1)
Omega-6	3.9 (90)	1.1 (25)	2.7 (26)	1.4 (14)	0.5 (5)
Lutein	1.0 (22)	0.1 (3)	2.5 (24)	0.7 (7)	0 (0)
Lycopene	0.1 (3)	0 (1)	0.5 (5)	0.1 (1)	0 (0)
Algae (spirulina, chlorella, kelp)	0.8 (17)	0.1 (2)	2.0 (20)	0.3 (3)	0 (0)
Ginseng	0.7 (16)	0.04 (1)	1.5 (15)	0.1 (1)	0 (0)
Citrus flavonoids	0.4 (10)	0.2 (4)	3.0 (29)	1.7 (17)	0.2 (2)
Grape seed extract	0.3 (7)	0.1 (3)	0 (0)	0 (0)	0.1 (1)
Ashwagandha	0.5 (11)	0.1 (2)	1.4 (14)	0.4 (4)	0.1 (1)
Horestail	1.5 (34)	0.3 (8)	1.7 (17)	0.8 (8)	0.1 (1)

Collagen	1.5 (35)	0.5 (12)	2.6 (25)	2.0 (20)	0.2 (2)
Q10 coenzyme	0.6 (14)	0.2 (5)	1.6 (16)	0.5 (5)	0.5 (5)
Propolis / pollen	0.2 (4)	0.2 (5)	0.5 (5)	0 (0)	0 (0)
Acerola	0.1 (2)	0 (0)	1.2 (12)	0.3 (3)	0 (0)
Protein / amino acids	1.8 (42)	0.4 (11)	1.1 (11)	0.8 (8)	0.1 (1)
Colostrum	0.2 (4)	0.3 (7)	0.3 (3)	0 (0)	0 (0)
Lactoferrin	0.04 (1)	0.04 (1)	0.2 (2)	0.2 (2)	0.1 (1)
Sea buckthorn	0 (0)	0 (0)	0.4 (4)	0.1 (1)	0 (0)

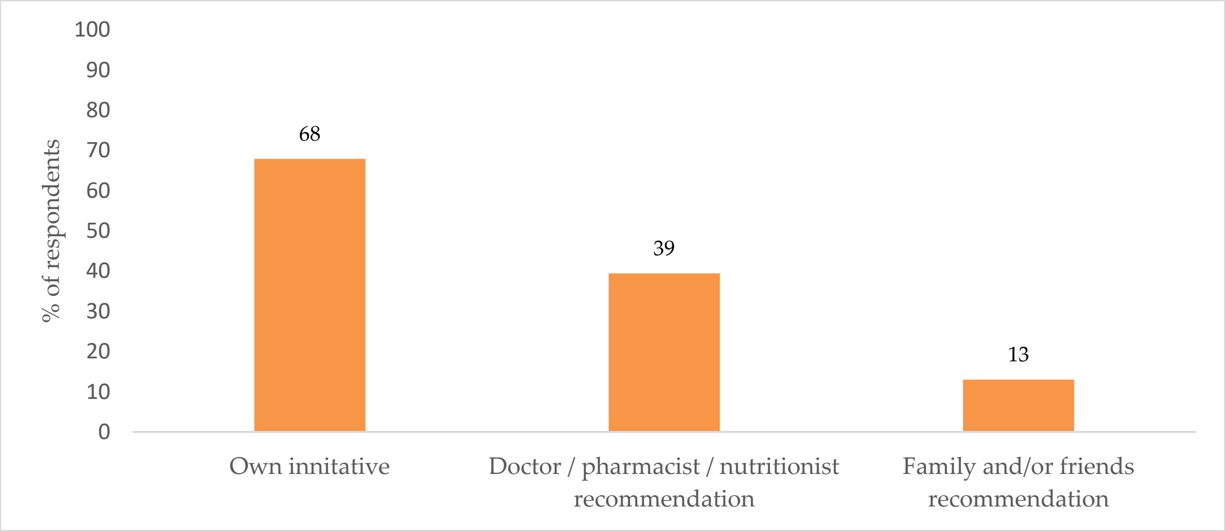

Figure S4. Source of recommendations of dietary supplements usage – results of PLifeCOVID-19 Online Study 2 (*n* = 978).

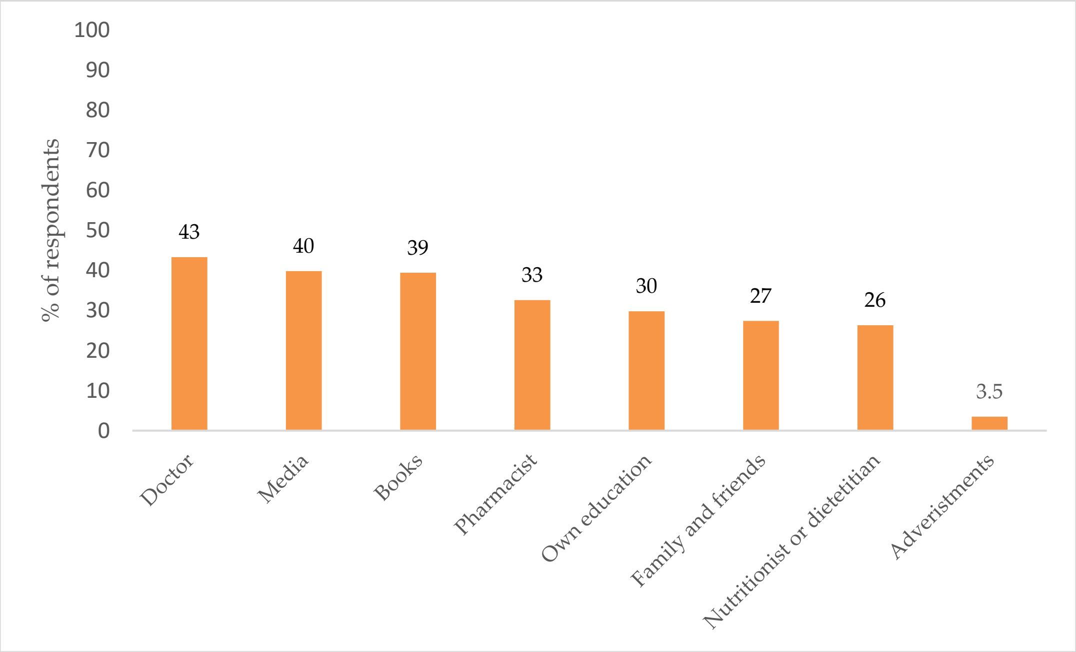

Figure S5. Source of knowledge about dietary supplements usage – results of PLifeCOVID-19 Online Study 2 (*n* = 978).

© 2020 by the authors. Licensee MDPI, Basel, Switzerland. This article is an open access article distributed under the terms and conditions of the Creative Commons Attribution (CC BY) license (http://creativecommons.org/licenses/by/4.0/).
